# A Qualitative Study of Perceived Barriers to Fruit and Vegetable Consumption Among Low-Income Populations, North Carolina, 2011

**DOI:** 10.5888/pcd10.120206

**Published:** 2013-03-14

**Authors:** Lindsey Haynes-Maslow, Sarah E. Parsons, Stephanie B. Wheeler, Lucia A. Leone

**Affiliations:** Author Affiliations: Sarah E. Parsons, Duke University, Durham, North Carolina; Stephanie B. Wheeler, Lucia A. Leone, University of North Carolina at Chapel Hill, Chapel Hill, North Carolina.

## Abstract

**Introduction:**

Obesity is the leading preventable cause of illness and a major contributor to chronic disease. Eating fresh fruits and vegetables can help manage and prevent weight gain and reduce the risk of chronic diseases. Low-income communities often lack stores that sell fresh fruit and vegetables and have instead stores that sell foods low in nutritional value. The objective of this study was to understand perceived community-level barriers to fruit and vegetable consumption among low-income people.

**Methods:**

We conducted 8 focus groups involving 68 low-income participants in 2 North Carolina counties, from May 2011 through August 2011. The socioecological model of health guided data analysis, and 2 trained researchers coded transcripts and summarized findings. Four focus groups were conducted in each county; 1 was all male, 5 all female, and 2 mixed sexes. Most participants were black (68%), most were women (69.1%), and most had a high school education or less (61.8%). Almost half received support from either the Supplemental Nutrition Assistance Program or another government assistance program.

**Results:**

We identified 6 major community-level barriers to access to fruits and vegetables: cost, transportation, quality, variety, changing food environment, and changing societal norms on food.

**Conclusion:**

Policymakers should consider supporting programs that decrease the cost and increase the supply of high-quality fruits and vegetables in low-income communities.

## Introduction

Obesity is the leading preventable cause of illness in the United States and a major contributor to chronic disease and health care costs (1). Eating healthful foods, including fresh fruits and vegetables, can help maintain weight and prevent weight gain and can reduce the risk of chronic diseases, including heart disease, diabetes, and cancer (1,2). Most people, especially low-income people, do not eat recommended amounts of fruit and vegetables (3,4).

Research addressing fruit and vegetable consumption among low-income populations often focuses on individual-level barriers such as cost, inadequate time for preparation (5), poor knowledge of nutrition (6), and limited cooking skills (7). However, focusing on individual-level barriers may obscure the role that community-level barriers, such as the food environment, may play in the socioeconomic disparities in both diet and obesity (8,9). Residents of low-income neighborhoods lack access to stores that sell healthful foods and are closer to convenience stores or fast food restaurants that sell food low in nutritional value (8,10,11). Even when healthful foods are available, low-income people often cannot afford them (12,13).

To date, most of the literature on food access has come from quantitative studies focusing on proximity and type of food stores available in the community (14,15). Qualitative research is needed to identify other access factors and gain a greater understanding of perceived barriers to eating fruit and vegetables. Although qualitative studies have been conducted on food access, they focus mostly on individual-level barriers (16–18) or communities in the North or Midwest (19–21). Our study is unique because it focuses on community-level barriers and because it was conducted in a state, North Carolina, that is ranked both in the top 10 agricultural states in the United States (22) and in the top 10 for food insecurity (23). North Carolina’s population is the 14th most overweight in the nation (1). Orange and Durham counties are a mix of urban and suburban communities. About 25% of adults in these 2 counties are obese, 16% are living in poverty, and 30% lack access to healthful foods (24).

To add to the qualitative literature on barriers to fruit and vegetable access, our study used focus groups to gain an understanding of perceived barriers to fruit and vegetable consumption among low-income people. The objective of this study was to examine how community-level characteristics affect individual dietary behaviors among low-income people by examining attitudes and beliefs about purchasing, preparing, and eating fruits and vegetables.

## Methods

### Recruitment

We obtained data by conducting 8 focus groups in 2 counties in North Carolina from May 2011 through August 2011. We excluded personal identifying data. This study was approved by the University of North Carolina’s institutional review board.

We used Internet searches to identify organizations that provide services to low-income residents in Orange and Durham counties (Table 1). We met with key informants in each of these organizations who could provide information about the dietary concerns of people served by the organization, recruit participants for focus groups by distributing informational flyers, and facilitate scheduling of focus groups. Key informants received $40 as compensation for their time.

**Table 1:** Focus Group Characteristics, Qualitative Study of Perceived Barriers to Fruit and Vegetable Consumption Among Low-Income Populations, North Carolina, 2011

County	Focus Group Site	Focus Group No. (No. of Participants)	Sex	Race	Site Description	Income Qualifier
Orange	Senior center	1 (11)	All female	Mixed race	Offers classes, wellness programs, trips, and lunches to residents aged 55 or older.	Open to seniors of all incomes. Researchers purposely targeted people using the senior center’s free lunch program^a^ but did not screen based on income
Orange	Senior center	2 (10)	All male	Mixed race	Located in a public housing community; offers classes and programs to neighborhood residents	Total annual household income cannot exceed 80% of the median household income set by the Department of Housing and Urban Development for the Chapel Hill, NC, area
Orange	Family resource center	6 (6)	All female	All black		
Orange	Family resource center	7 (8)	All female	All black	Located in low-income neighborhood; offers after-school classes for children and teens	Located in a census tract in which the median annual household income is less than $27,550
Durham	Community center	5 (6)	All female	All black		
Durham	Recovery shelter	4 (10)	Mixed sex	Mixed race	Located in low-income neighborhood; offers 6-month, live-in drug and alcohol rehabilitation program for homeless adults	Located in a census tract in which the median annual household income is less than $27,550
Durham	Small grocery store	3 (9)	Mixed sex	All black	Located in a low-income neighborhood; owned and operated by a nonprofit that provides work-based vocational training for recovering substance abusers	Located next to 3 census tracts in which the median annual household income is less than $27,550
Durham	Latino resource center	8 (8)	All female	All Latina	Offers programs, education, and leadership development to Latinos/Hispanics in the area	Open to Latinos/Hispanics of all incomes

a The free lunch program is a federally funded program for seniors aged 60 or older.

### Focus groups

Focus groups met at the same community facilities where participants were recruited. Inclusion criteria were being aged 18 years or older and having been identified by a key informant as low-income (Table 1). We recruited a total of 68 participants with 6 to 11 in each group. Members of 5 of the focus groups were predominately black; 2 comprised elderly men and women (≥57 y), and 1 was entirely Latina. Participants provided informed consent and completed a demographic survey. Each focus group met for 60 minutes.

Focus groups were moderated by 2 lead authors (L.H.M. and S.E.P.), each with 5 years of experience in qualitative research. A native Spanish speaker trained in qualitative research methods conducted the Latina focus group in Spanish. Moderators had no prior relationship with participants. We provided moderators with a focus group guide to elicit discussion about participants’ ability to buy fresh fruits and vegetables instead of canned, frozen, or fried; current grocery shopping behaviors; perceived barriers to accessing fruits and vegetables in their community; and strategies to improve access in their community (Appendix). Focus group discussions were recorded. Participants received $25.

### Data analysis

Focus group sessions were transcribed verbatim and imported into Atlas.ti 6.0 (Atlas.ti Scientific Software Development, Berlin, Germany). The 2 lead authors read transcripts multiple times before coding and analysis. Analysis was guided by the socioecological model of health, which posits that health and health behaviors are influenced at 4 levels: individual (ie, genetics, demographics, and personal health beliefs); interpersonal (ie, family, friends, and peers); community (ie, social networks, norms, environmental characteristics such as access to and quality of fruits and vegetables, and transportation infrastructure); and societal (ie, public policies and systems) (25). During coding, researchers focused on community-level determinants of fruit and vegetable consumption behaviors including how access is affected by availability, affordability, and quality of fruits and vegetables; city design and transportation infrastructure; and societal norms.

In the initial coding phase, the 2 lead authors independently applied open coding to 2 transcripts to identify topics and issues raised. We compared open codes, reconciled discrepancies through discussions, and revised the code book. The revised code book was independently applied to all 8 focus groups. During the analysis phase, we conducted in-group analysis of the major themes and issues for each focus group and wrote memos summarizing findings from each group. Memos were compared across all focus groups to ensure data saturation had been reached.

## Results

Most participants were black (67.7%) and female (69.1%) with a high school education or less (61.8%) (Table 2). Almost half received support from Supplemental Nutrition Assistance Program (SNAP) or another government assistance program. Most participants shopped at supermarkets and supercenters (eg, Walmart). Several participants shopped at a small, locally owned grocery store in their neighborhood. Additionally, women in the Latina focus group shopped for fruits and vegetables at a flea market where most vendors spoke Spanish and sold culturally appealing produce, such as guava, mangos, and plantains.

**Table 2:** Demographic Characteristics of Focus Group Participants (N = 68), Qualitative Study of Perceived Barriers to Fruit and Vegetable Consumption Among Low-Income Populations, North Carolina, 2011

Characteristic	n (%)^a^
**Age, y**	
20–29	9 (13.2)
30–39	11 (16.2)
40–49	9 (13.2)
50–59	16 (23.5)
60–69	9 (13.2)
≥70	12 (17.6)
Did not answer	2 (2.9)
**Sex**	
Female	47 (69.1)
Male	21 (30.9)
**Race/ethnicity**	
White	14 (20.6)
Black	46 (67.7)
Latino	8 (11.7)
**Education**	
8th grade or less	8 (11.8)
Some high school	11 (16.2)
High school or GED diploma	23 (33.8)
Some college	16 (23.5)
College degree or more	10 (14.7)
**Annual household income, $**	
<10,000	32 (47.0)
10,000–19,999	18 (26.4)
20,000–29,999	8 (11.7)
≥30,000	4 (5.9)
Did not answer	6 (8.8)
**Marital status**	
Not married	29 (42.6)
Married/living with partner	19 (27.9)
Separated	5 (7.4)
Divorced	14 (20.9)
Did not answer	1 (1.2)
**Receive SNAP benefits**	
Yes	31 (45.6)
No	37 (54.4)
**Receive WIC, TANF, Medicaid, Work First, or other assistance**	
Yes	31 (45.6)
No	32 (47.1)
Did not answer	5 (7.3)

Abbreviations: GED, general equivalency degree; SNAP, Supplemental Nutrition Assistance Program; WIC, Special Supplemental Food Program for Women, Infants and Children; TANF, Temporary Assistance for Needy Families.

a Percentages do not add to 100% because of rounding.

We identified 6 major community-level barriers affecting access to fruit and vegetables: cost, transportation, quality, variety, changing food environment, and changing societal norms (Table 3).

**Table 3:** Focus Group Barriers and Code Frequencies, Qualitative Study of Perceived Barriers to Fruit and Vegetable Consumption Among Low-Income Populations, North Carolina, 2011

Barriers	Illustrative Quote	No. of Focus Groups That Referenced Barrier	No. of References Across All Focus Groups^a^
Cost	“Just based on what I’ve seen here in the Durham area and in the Carolinas, in general, I can’t believe that they grow so much fruits and vegetables, and you can’t afford them! Up north, it’s different. At times the prices go up on fruits and vegetables, depending on the crops. But, basically, the fruits are cheaper, and it’s just kind of ironic to me to be in the South and see that you can’t afford hardly to eat the produce. It’s just really strange.” (black female, focus group 5)	8	103
Transportation	“I go on the bus . . . sometimes someone gives me a ride, but mostly I go on the bus. I make the trip by parts, because I cannot take many things [on the bus].” (Latina female, focus group 8)	8	26
Quality	“You have to go way into an upper-class neighborhood in order to get fruits and vegetables that actually look good enough to consume.” (black female, focus group 3)	8	26
Variety	“As far as having a variety of what you really want . . . no. Not in this community.” (black male, focus group 3)	7	10
Changing food environment	“When I was growing up, I never remember canned goods being in our house. Everything was fresh, because the vegetable man came every day!” (black female, focus group 6)	8	28
Changing societal norms	“The women don’t want to cook no more now. They’ll tell you right quick, ‘We’re going out to eat. I ain’t cooking nothing.’ So you don’t find women like my mother, my mother with 15 meals a day, and you come home and there was always a good meal waiting for them. But now, your wife look at you like, ‘I don’t feel like messing in no kitchen.’” (black male, focus group 1)	6	18

a Total references were calculated based on the number of times participants made statements that were coded as that barrier.

### Cost

Cost was cited 103 times among all focus groups and was the most commonly and extensively described barrier to purchasing fresh fruits and vegetables. Participants talked about cost 4 times as often as any other barrier. Even when participants were discussing other barriers to fruit and vegetable consumption, cost was cited. Most participants expressed frustration with not being able to buy and prepare as many fruits and vegetables as they would like for themselves and their family:

What we need to eat — and what we want to eat — the price is a big part of it. When you have lower-income families, they usually don’t introduce fruit and vegetables into their children’s body because it costs so much. So, if there were . . . if there was a price where everybody could afford it, then everybody could have it. (black female, focus group 3)

In half of the focus groups, participants expressed a preference for fresh fruits and vegetables; however, some women reported buying more canned or frozen fruits and vegetables because they were cheaper.

### Transportation

Transportation was another common barrier to purchasing fruits and vegetables, especially for the elderly or those who did not own a vehicle. Some participants mentioned that the lack of transportation options made it difficult to travel to other parts of the city, including farmers markets, to access high-quality produce. One elderly participant commented, “I’d love to go to Whole Foods, you know . . . [but] I can’t afford a car anymore. I could drive. I have vision, I’m capable. But I can’t afford the insurance, and I don’t drive.” (white male, focus group 2)

Participants who did not own a car relied on riding the bus, walking, or carpooling. Riding the bus and walking limited the number of grocery bags they could carry home. Buses created problems because of the need to transfer, inconvenient schedules, and bus stop locations. Most Durham participants mentioned safety and risk of groceries and purses being stolen as an issue when walking home with bags of groceries. In the Latina focus group, women discussed traveling longer distances to a flea market to purchase their fresh fruits and vegetables. The flea market generally had lower prices and sold fruits and vegetables that they were accustomed to cooking in their native countries.

### Quality

Most participants preferred high-quality fruits and vegetables but reported a lack of quality in their area. Participants reported being less likely to purchase fruits and vegetables from stores that did not market or display their produce in an appealing manner. Several participants acknowledged that they wanted to support a locally owned grocery store in their neighborhood but that the quality of produce discouraged them from doing so: “I tell them, “Look, these apples are rotten.” They’re pretty on the outside, but they’re rotten. So they gave me another one . . . that was rotten, as well.” (black female, focus group 3)

Another participant said the only place in his neighborhood he could purchase high-quality fruits was at a corporate-owned gas station. He felt guilty shopping there but felt quality was a higher priority than supporting local businesses.

### Variety

Most participants reported that the grocery stores in their communities where they shopped most frequently lacked a variety of fruits and vegetables. In 1 Durham focus group, participants described how they saw the same limited selection of fruits and vegetables each week and that to find more variety they had to go to a farmers market. In Orange County, 1 woman reported having similar experiences: “You can get vegetables but not the variety of vegetables that you might want” (white female, focus group 1).

Limited selection decreased participants’ willingness to shop at grocery stores, but participants with limited transportation found it difficult to search for fruits and vegetables elsewhere.

### Changing food environment

In the past several decades, there have been vast changes to the food environment (eg, grocery stores, supermarkets, farmers markets, roadside stands) in low-income neighborhoods. Residents have limited access to farm produce, and fast food restaurants proliferate. Some participants talked about the good old days when they walked down their neighborhood street and purchased produce directly from farmers: “He had an old truck and he sold vegetables out of his truck . . . for me, I don’t see him no more . . . but that would be nice if we had somebody who would come around with vegetables.” (black female, focus group 6)

Fast food restaurants were recognized as being part of the current food environment. Reviews were mixed on the affordability of such restaurants — some participants thought they were affordable while other participants did not. Participants associated consumption of unhealthful fast food with obesity in America:

I think that another problem with America and obesity is the fact that people do not get enough fruits and vegetables. Therefore, a lot of people rely on, “If I only got a dollar, I’ll just [go] to McDonald’s and get a double cheeseburger,” because that’s going to work. I think that’s contributing to the obesity in the population as a whole, simply because [of] the food prices. (black female, focus group 5)

Despite fast food being viewed as unhealthful and linked with obesity, participants still purchased it because it was close to their homes and therefore convenient.

### Changing societal norms

In all 8 focus groups, participants seemed to recognize the changing societal norms on food in the United States: the social shifts of the role of women in the family, children having more choice, and the overall shift from less emphasis on cooking and more emphasis on convenience. Although the change in societal norms is not specific to low-income North Carolinians, we wanted to understand its effect on our participants. All focus group participants discussed food preparation time and convenience of prepared food as barriers to eating fruits and vegetables. Several men in the senior center’s men’s focus group believed that having women in the workforce decreased the quality and nutritional value of meals served at home.

Women don’t cook. It’s convenient [going out to eat], and then they don’t cook. People working 2 and 3 jobs, don’t want to do it. . . . See, back when I was growing up, my mother didn’t work. So she’d stay home and cook. But most of the women now work. And they say, “I ain’t cooking, I worked all day.” They say, “We’ll go out and eat.” (black male, focus group 2)

### Other societal issues

Although the effect of women in the workplace on eating out did not explicitly emerge as a theme in other focus groups, a woman in 1 group stated:

I can cook with fresh vegetables. I felt like it just took too much time that I didn’t have when I was working. But now that I’m not working . . . not that I’m using it for an excuse, but now that I have more time I go into the kitchen and I’m like [mimics chopping vegetables] and I just throw all of it together. (black female, focus group 7)

Several women talked about the lack of time during the day to cook, the convenience of prepared or fast foods, and the struggle to provide food that their children liked. Often, women talked about ceding to their children’s food preferences to avoid wasting time on arguments. When discussing the topic of why children were not eating as many fruits and vegetables, 1 mother said it was partially her fault and that she did not make her kids eat foods they disliked. The senior center men’s focus group also raised the same topic, and 1 man commented, When we was growing up, you ate what was put on the table. You weren’t asked what you wanted to eat.” (black male, focus group 2)The Latina focus group also talked about purchasing more fast foods because their children requested it and because fast food was convenient for days when they were in a hurry.

## Discussion

Participants identified 6 main barriers to eating fruits and vegetables: cost, transportation, lack of quality and variety, changing food environment, and changing societal norms. Our findings suggest that quality and variety are important barriers to fruit and vegetable consumption. To date, the literature on the relationship between access to fruits and vegetables and consumption is mixed. In a longitudinal study of 5,000 adults, access to more supermarkets was unrelated to fruit and vegetable consumption (26). However, another study found that with each additional supermarket in a census tract, fruit and vegetable consumption among black residents increased by 32% (11). As conveyed by our participants, proximity to fresh fruits and vegetables may be a necessary, but not sufficient, facilitator to increasing consumption. A quantitative study of 495 residents in 6 low-income communities in Chicago, Illinois, also found that quality, selection, and convenience were factors associated with greater consumption of fruits and vegetables (27).

In all focus groups, participants expressed a desire for increased quality and variety of fresh fruits and vegetables conveniently located in their community. Often, grocery stores or farmers markets tend not to locate in low-income communities because of perceived lack of demand (27), and when they do, quality of their fruits and vegetables tends to be poorer than in higher-income neighborhoods. A recent study in Connecticut found that low-income neighborhoods had lower quality fresh fruits and vegetables than higher-income neighborhoods (28).

Many participants said they wished that farmers could drive to their houses to sell produce from trucks or operate roadside stands near their communities. If outlets with fresh fruits and vegetables resembled the convenience of fast food restaurants, people may be more likely to eat them. These results mirror findings from a recent survey of low-income people in which the most frequently cited facilitator to eating more fruits and vegetables was easy access to affordable, locally grown produce (5). Although farmers markets have been cited as 1 method for increasing convenience of purchasing fruits and vegetables, many focus group participants discussed barriers to shopping there, including location and inconvenient hours.

Our study has several limitations. First, the small sample size and narrow geographic location limits generalizability of our findings. Second, low-income people who choose to participate in focus groups may have different barriers to fruit and vegetable consumption than those who do not. Nonparticipants may be eating fewer fruits and vegetables and may face greater barriers. We plan to address these limitations in future studies by conducting more focus groups in other North Carolina counties.

Our study increases understanding of the experience of low-income North Carolinians that may be relevant to other settings. Reducing cost and improving quality, variety, and convenience of fresh fruits and vegetables may increase consumption and reduce chronic disease. Policy makers should consider supporting programs that address these barriers in low-income communities. This may be done by encouraging grocery stores to locate in these communities, creating incentives for convenience stores to carry more produce, and restricting the number of fast food restaurants that can locate in these communities. Additionally, policy makers should consider decreasing fruit and vegetable prices, either through subsidies or vouchers, to address cost barriers. Policy makers should invest in culturally appropriate nutrition programs that focus on increasing fruit and vegetable consumption among low-income people.

## Appendix. Focus Group Questions

What’s your name and what brings you here today?

Why do you eat fruits and vegetables?

*     Probe: General health, weight management*

Are you able to buy and prepare as many fruits and vegetables as you would like for yourself or your family?

What makes it harder?

What would make it easier?

*     Probe: Distance, knowledge of how to prepare foods, cooking equipment*

Where do you most often buy fresh fruits and vegetables?

Why do you buy fruits and vegetables at this location?

What is most important to you when choosing fruits and vegetables?

*     Probe: How important is it that your produce be from North Carolina farms?*

How important is it that your produce be organic or grown without chemicals or pesticides?

Would you like to see more options in your community for purchasing fresh fruits and vegetables?

What types of programs would help you to eat more fruits and vegetables?

Is there anything else you would like to share with us?
